# Hu Antigen R (HuR) Protein Structure, Function and Regulation in Hepatobiliary Tumors

**DOI:** 10.3390/cancers14112666

**Published:** 2022-05-27

**Authors:** Sofia Lachiondo-Ortega, Teresa Cardoso Delgado, Blanca Baños-Jaime, Alejandro Velázquez-Cruz, Irene Díaz-Moreno, María Luz Martínez-Chantar

**Affiliations:** 1 Liver Disease Lab, Center for Cooperative Research in Biosciences (CIC bioGUNE), Basque Research and Technology Alliance (BRTA), 48160 Derio, Spain; slachiondo@cicbiogune.es (S.L.-O.); tcardoso@cicbiogune.es (T.C.D.); 2 Centro de Investigaciones Científicas Isla de la Cartuja (cicCartuja), Instituto de Investigaciones Químicas (IIQ), Universidad de Sevilla, Consejo Superior de Investigaciones Científicas (CSIC), 41092 Sevilla, Spain; blanca.banos@iiq.csic.es (B.B.-J.); alejandro.velazquez@iiq.csic.es (A.V.-C.); idiazmoreno@us.es (I.D.-M.); 3 Centro de Investigación Biomédica en Red de Enfermedades Hepáticas y Digestivas (CIBERehd), Carlos III National Health Institute, 28029 Madrid, Spain

**Keywords:** ELAV-like protein 1, RNA-binding protein, hepatocellular carcinoma, cholangiocarcinoma

## Abstract

**Simple Summary:**

Hepatobiliary tumors are a group of primary malignancies encompassing the liver, the intra- and extra-hepatic biliary tracts, and the gall bladder. Within the liver, hepatocellular carcinoma (HCC) is the most common type of primary cancer, which is, also, representing the third-most recurrent cause of cancer-associated death and the sixth-most prevalent type of tumor worldwide, nowadays. Although less frequent, cholangiocarcinoma (CCA) is, currently, a fatal cancer with limited therapeutic options. Here, we review the regulatory role of Hu antigen R (HuR), a ubiquitous member of the ELAV/Hu family of RNA-binding proteins (RBPs), in the pathogenesis, progression, and treatment of HCC and CCA. Overall, HuR is proposed as a valuable diagnostic and prognostic marker, as well as a therapeutic target in hepatobiliary cancers. Therefore, novel therapeutic approaches that can selectively modulate HuR function appear to be highly attractive for the clinical management of these types of tumors.

**Abstract:**

Hu antigen R (HuR) is a 36-kDa ubiquitous member of the ELAV/Hu family of RNA-binding proteins (RBPs), which plays an important role as a post-transcriptional regulator of specific RNAs under physiological and pathological conditions, including cancer. Herein, we review HuR protein structure, function, and its regulation, as well as its implications in the pathogenesis, progression, and treatment of hepatobiliary cancers. In particular, we focus on hepatocellular carcinoma (HCC) and cholangiocarcinoma (CCA), tumors where the increased cytoplasmic localization of HuR and activity are proposed, as valuable diagnostic and prognostic markers. An overview of the main regulatory axes involving HuR, which are associated with cell proliferation, invasion, metastasis, apoptosis, and autophagy in HCC, is provided. These include the transcriptional, post-transcriptional, and post-translational modulators of HuR function, in addition to HuR target transcripts. Finally, whereas studies addressing the relevance of targeting HuR in CCA are limited, in the past few years, HuR has emerged as a potential therapeutic target in HCC. In fact, the therapeutic efficacy of some pharmacological inhibitors of HuR has been evaluated, in early experimental models of HCC. We, further, discuss the major findings and future perspectives of therapeutic approaches that specifically block HuR interactions, either with post-translational modifiers or cognate transcripts in hepatobiliary cancers.

## 1. Introduction

Hu antigen R (HuR), also known as HuA or embryonic lethal abnormal vision-like protein 1 (ELAVL1), is a 36-kDa ubiquitous member of the ELAV/Hu family of RNA-binding proteins (RBPs), which, also, includes HuB, HuC and HuD [1,2]. In humans, this protein is encoded by the ELAVL1 gene. In healthy individuals, HuR protein is, mostly, expressed in human endocrine tissues, the respiratory system, and the gastrointestinal tract [3].

### 1.1. HuR Protein Structure

**Figure 1:**
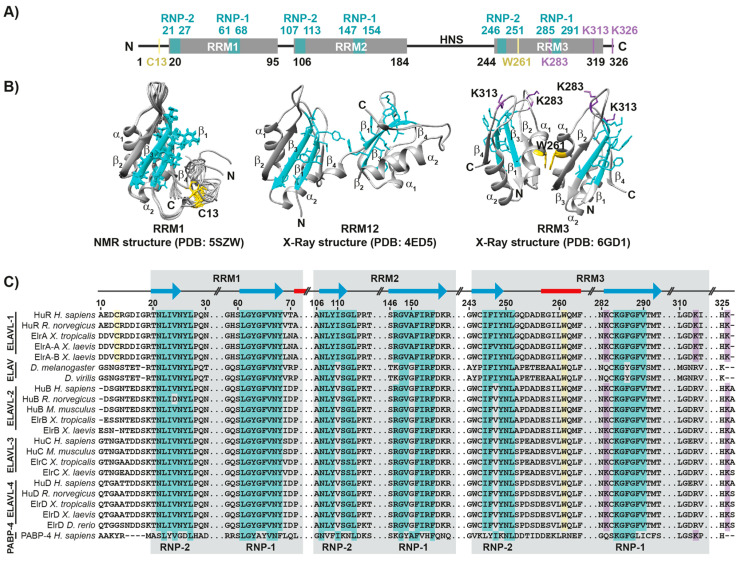
HuR domain organization, structures, and sequence alignment. (**A**) Schematic domain organization of human HuR. Black numbers indicate the residues flanking each domain. RNPs and residues flanking RNPs are colored in cyan. C13 and W261, implicated in dimerization, are highlighted in yellow. Susceptible lysines to become NEDDylated are highlighted in purple. N and C indicate N-terminal and C-terminal endings, respectively. (**B**) Ribbon structures of human HuR RRM domains. Side-chains of residues at RNPs are colored in cyan, those of C13 and W261 are highlighted in yellow; NEDDylated lysines are shown in purple. β-strands and α-helixes numbers are indicated. Left panel: NMR structure of RRM1 domain. Ten models are shown (PDB: 5SZW) [14]. Middle panel: X-ray structure of RRM12 tandem of HuR (PDB: 4ED5) [5]. Right panel: X-ray structure of RRM3 domain, in its dimeric form (PDB: 6GD1) [15]. (**C**) Sequence alignment of HuR, with homologous RNA binding proteins (ELAV, ELAVL-1, ELAVL-2, ELAVL-3, ELAVL-4, and PABP-4), from human and related organisms. Residues belonging to each RRM domain are shaded in grey. RNPs residues from HuR are highlighted in cyan, whereas C13 and W261 are in yellow. K283, K313, and K326 are highlighted in purple. Blue arrows represent β-strands, and red rectangles represent α-helixes of HuR.

HuR structure is, mainly, comprised of three RNA-recognition motifs (RRMs) (Figure 1A) [4]. The first two domains (RRM1 and RRM2) are connected by a 10-residue linker in a tandem structure, as revealed by its X-ray crystallographic model (Figure 1B, middle panel), and are the main domains involved in RNA recognition [5]. Despite the high dynamics of the N-terminal unstructured 20-residue stretch of HuR, nuclear magnetic resonance (NMR) experimental restraints revealed that it was folded towards the RRM1 β-sheet (Figure 1B, left panel) [6]. Interestingly, it harbors the HuR redox sensor Cys13, which is conserved among ELAVL1 orthologous proteins and is involved in HuR RRM1 and RRM1-2 homodimerization and, to some extent, in the regulation of the antioxidant response by HuR (Figure 1C) [7,8]. A 60-residue disordered hinge region spaces out the C-terminal RRM3 domain from the tandem RRM1-2. This linker region contains the HuR nucleocytoplasmic shuttling sequence (HNS) (Figure 1A), whose length enables transient contacts between RRM3 and RRM1–2 [9]. Phosphorylation at HNS modifies HuR interaction with nucleo-cytoplasmic transport proteins, triggering HuR accumulation in the cytosol [10,11]. HuR RRM3, also, forms dimers through its evolutionary well-conserved Trp261 residue along evolution (Figure 1C), which is located in helix α1 of RRM3 (Figure 1B, right panel) [12,13].

All three RRMs show the canonical β_1_α_1_β_2_β_3_α_2_β_4_ topology, with a β-sheet formed by four antiparallel β-strands folded against two α-helices. The two central β-strands (β_1_ and β_3_) of each RRM contain the ribonucleoprotein (RNP) motifs RNP-1 (in β_3_) and RNP-2 (in β_1_), which are essential for RNA/DNA binding (Figure 1B,C) [15,16]. HuR RRM domains bind single-stranded RNA/DNA, by means of stacking interactions and hydrogen bonds between RNP motifs and nucleic acids [17]. RNP motif sequences are indicated in Figure 1C and, even though they differ for each RRM of HuR, they have been extensively conserved in ELAV-like proteins, through evolution from *Drosophila* to vertebrates [18].

### 1.2. HuR Protein Function

HuR functions as a post-transcriptional regulator, affecting many aspects of RNA metabolism, from splicing to translation, by binding through its RRMs to AU-rich elements (AREs), typically present in the 3′-untranslated region (UTR) of its target transcripts [19,20]. Under physiological conditions, HuR is, predominantly, located in the nucleus, where it participates in pre-mRNA splicing and nuclear export of mature mRNAs [21,22,23,24,25]. Notably, its nuclear functions remain poorly understood. Upon specific stimuli (such as stress signals and mitogens), HuR translocates to the cytoplasm and develops its main biological functions, which are mRNA stabilization and modulation of translation. HuR nucleo-cytoplasmic shuttling, through the nucleopore, is aided by the import factors transportin-1 (Trn1) and -2 (Trn2), and the adaptor proteins ANP32A (pp32/PHAP-I) and ANP32B (APRIL), both independently mediating HuR association with the nuclear export receptor chromosome region maintenance 1 (CRM1) [22,26,27].

In the cytoplasm, it is thought that HuR might control RNA stability, by competing with other RBPs and, thus, preventing mRNA degradation and deadenylation. To date, over 80 HuR-stabilized target mRNAs have been described and reviewed in [28,29], which include those that encode c-Fos [30], p21 [31,32,33], cyclins A2 [30,34], B1 [30,34], E1 [35,36], D1 [30,37], inducible nitric oxide synthase (iNOS) [38,39], granulocyte macrophage-colony stimulating factor (GM-CSF) [40,41], eukaryotic initiation factor (eIF)-4E [42], murine double minute (Mdm)2 [43,44], vascular endothelial growth factor (VEGF) [45,46,47,48,49,50], transforming growth factor (TGF)-β [45], sirtuin 1 (SIRT1) [51,52], tumor necrosis factor (TNF)-α [40,45,53], B-cell leukemia (Bcl)-2 [54,55], myeloid leukemia cell differentiation protein (Mcl)-1 [54], oncostatin M (OSM) [56], cyclooxygenase (COX)-2 [30,45,52,57,58,59,60,61,62], γ-glutamylcysteine synthetase heavy subunit (γ-GCSh) [63], survival of motor neuron (SMN) [64], SH2D1A [65], the regulator of G-protein signaling 4 (RGS4) [66], parathyroid hormone-related protein (PTHrP) [67], Fas ligand (FasL) [68], Myogenin [69,70], MyoD [69,70], acetylcholinesterase (AChE) [71,72], p53 [73,74], ARHI [aplasia Ras homolog member I (DIRAS3)] [58], nitric oxide/soluble guanylyl cyclase (sGC) [75], urokinase plasminogen activator (uPA) and its receptor (uPAR) [76], neurofibromatosis type 1 (NF1) [77], von Hippel-Lindau protein (pVHL) [67,78], toll-like receptor 4 (TLR4) [79], Snail [80], matrix metalloprotease (MMP)-9 [81,82,83,84,85], c-Fms [86], the MAPK phosphatase (MKP)-1 [87], interferon (IFN)-γ [88], interleukin (IL)-3 [89], IL-4 [90], IL-6 [45,91,92], IL-8 [45,57,93], and HuR itself [25,94,95].

Regarding the control of translation, HuR can promote the expression of many mRNAs that are templates for proteins, such as cyclin A2 [30,34,96], prothymosin α (ProTα) [97], hypoxia-inducible factor (HIF)-1α [98], Bcl-2 [54,55,99], VEGF [45,46,47,49,100,101,102], thrombospondin (TSP)-1 [103], MKP-1 [87], p53 [73,74], the cationic amino acid transporter (CAT)-1 [104], the intrinsic cellular caspase inhibitor XIAP [37], and cytochrome c [105]. Importantly, HuR can also bind to the 5′-UTR of a small subset of target transcripts (e.g., p27, IGF-1R and thrombomodulin) and disrupt the internal ribosome entry site (IRES)-dependent translation, eventually resulting in the repression of protein expression [106,107,108]. HuR was, also, found to bind to the 3′-UTR of Wnt5a and c-Myc mRNAs and repress their translation [109,110].

In addition to coding transcripts, HuR has the ability to bind and regulate the function of microRNAs (miRNAs) [111,112,113,114] and long non-coding RNAs (lncRNAs) [111,115,116,117].

### 1.3. Regulation of HuR Protein Function

The function of HuR is regulated through transcriptional, post-transcriptional, and post-translational mechanisms, as discussed below.

#### 1.3.1. Transcriptional Control of HuR

The ELAVL1 gene displays alternate transcriptional start sites, leading to at least two distinct transcripts with distinct 5′-UTRs [99,118], the shorter of them being expressed in stress conditions under the influence of bone morphogenetic protein-7 (BMP-7) and the Smad 1/5/8 route [118]. Expression of the long HuR isoform relies on nuclear factor k of activated B-cells (NF-kB). The HuR promoter region contains a target of NF-kB, to which one of its members—p65/RelA—binds upon the induction of the phosphatidylinositol 3-kinase (PI3K)/AKT signaling route, resulting in the abnormal upregulation of HuR gene expression in gastric tumors [119].

#### 1.3.2. Post-Transcriptional Control of HuR

The stability of HuR mRNA is another key factor controlling its protein levels. HuR can both up- and down-regulate its expression, by interacting with its own transcript [25,94,95,120]. In addition, the existence of both positive and negative feedback loops between HuR and the protein product of its target mRNAs have been reported [121,122,123,124].

HuR abundance can, also, be regulated by miRNAs. An assortment of miRNAs (miR-125 [125], miR-519 [126], miR-9 [127], 291b-3p [128], miR-570-3 [129], miR-16 [130], and miR-22 [131]) has been found to target the coding region and 3′-UTR of *HuR* mRNA, suppressing its expression. Conversely, HuR 3′-UTR comprises a binding site for miRNA-155-5p, which stabilizes the transcript and favors HT-29 colorectal cancer (CRC) cell migration [132].

#### 1.3.3. Post-Translational Control of HuR

Post-translational modifications (PTMs) account for the main mechanism of regulation for HuR function, allowing the protein to elicit quick changes in gene-expression programs [10,11]. HuR protein abundance, subcellular localization, and RNA-binding affinity can be modulated by methylation [133,134,135,136], phosphorylation [24,33,44,51,137,138,139,140,141,142,143,144,145,146,147,148,149,150,151,152,153,154,155,156,157], proteolytic cleavage [158,159,160], ubiquitination [78,147,161,162], PARylation [163,164], sulfhydration [165], and arginylation [166].

More recently, it was described that circular RNAs (circRNAs) may comprise various binding motifs for one or several RBPs, including HuR, acting as sponges, where the RBP is prevented from binding to other targets while tethered to the circRNA [167]. For instance, it was shown that circPABPN1 competes with *PABP1* mRNA for binding to HuR, affecting the expression of p53, c-myc, and bcl-2, besides PABP1 [168].

#### 1.3.4. HuR Protein Oligomerization

HuR and functionally related proteins, such as AUF1, form dimers and multimers both free and bound to target sequences [12,15,153,169,170,171]. HuR dimers have been detected by fluorescence resonance energy transfer (FRET), both in the cell nucleus and cytoplasm. Importantly, the HuR oligomerization state strongly affects the stability of its target mRNAs. Fluorescence anisotropy analyses suggested that HuR RRM1-2 is able to form dimers with high affinity (*K*_D_ ca. 0.1 nM). These data indicate that RRM1-2 dimerization could be an important feature of the interaction mechanism between HuR and its target RNAs [172]. Furthermore, a structural analysis revealed that RRM1 alone forms homodimers as well, since Cys13 is exposed to and capable of establishing disulfide bonds, thus enabling HuR to respond to oxidative stress [7]. Beyond dimerization, cytoplasmic multimerization of HuR has been observed in glioma cells. A thorough analysis using distinct HuR constructs bound to fluorescent and bioluminescent probes enabled a model to be constructed, in which Cys13 is responsible for covalent dimerization, although the hinge region and RRM3 are still necessary for further oligomerization [173]. Indeed, the C-terminal domain of HuR is, also, prominently involved in its dimerization [12,13,15,170], as well as in the cooperative formation of HuR oligomers on ARE motifs [12,153,170]. According to small-angle X-ray scattering (SAXS) and NMR data, a single site-directed mutation (W261E) in RRM3 was enough to hinder its dimerization, under reducing conditions [15]. Moreover, deletion of RRM3 critically impaired the ability of the RBP to stabilize reporter β-globin mRNA constructs containing different AREs [9]. Altogether, experimental evidence points to HuR dimerizing through RRM1 and RRM3 in an independent way, while requiring both domains for oligomerization.

## 2. HuR and Hepatobiliary Cancers

HuR owns the post-transcriptional control of a large number of RNAs, enabling the protein to play pivotal roles that are dictated by the molecular functions of the transcripts. HuR targets, chiefly, include many mRNAs encoding proteins involved in cell proliferation, senescence, apoptosis, differentiation, stress, and immune responses. In turn, HuR was found to be involved not only in physiological processes (e.g., adipogenesis and muscle differentiation) [69,174,175,176] but also in disease (primarily, cancer and inflammation) [28,29,177]. Indeed, the relevance of HuR in some types of hepatobiliary tumors has been constantly described during the last decade. The major findings to date and future perspectives on the applications of HuR as a protein marker for the diagnosis, prognosis, and therapy of hepatobiliary cancers are the main topic of this review.

### 2.1. Hepatobiliary Cancers

Hepatobiliary cancers are a group of primary malignancies encompassing the liver, the intra- and extra-hepatic biliary tracts, and the gall bladder. Within the liver, the most common primary cancer is hepatocellular carcinoma (HCC). HCC, usually, arises in patients with an underlying chronic liver disease, such as chronic infection with the hepatitis B (HBV) or C virus (HBC), cirrhosis, excessive alcohol consumption, metabolic syndrome, diabetes, and non-alcoholic fatty liver disease (NAFLD) [178]. Even though the clinical management of HCC has improved in the past ten years, particularly for patients at advanced stages, HCC is still the third-most common cause of cancer-associated death and the sixth-most prevalent type of cancer worldwide [179]. Of relevance, in the past few years, important studies on immunotherapy in HCC have been carried out, resulting in novel guidelines for its treatment [180,181,182].

On the other hand, cholangiocarcinoma (CCA) encompasses a group of malignancies, arising at any point in the hepatic biliary tree. Biliary tract cancers, including intrahepatic (ICC), perihilar (PCC), and distal cholangiocarcinoma (DCC), as well as gallbladder cancer, are low-incidence malignancies in most high-income countries, but represent a major health problem in endemic areas. Indeed, ICC is the second-leading cause of primary liver cancer [183]. Nowadays, CCA is a fatal cancer, with a survival rate beyond a year of diagnosis inferior to 5% [184]. Therapeutic options are limited and surgery is the cornerstone of cure for CCA, even though most patients present with locally advanced or metastatic disease [185]. In the past few years, druggable alterations such as fibroblast growth factor receptor 2 (FGFR2) gene fusions and rearrangements, or isocitrate dehydrogenase-1 (IDH-1) and BRAF mutations, have been widely described in CCA patients, further indicating the important differences between iCCA and PCC/DCC, as thoroughly reviewed in [186].

### 2.2. HuR as a Prognostic and Diagnostic Biomarker in Hepatobiliary Cancers

HuR protein is, either, overexpressed in most human cancers or overactivated, as denoted by its increased cytoplasmic localization and the translation of various mRNAs involved in carcinogenesis. Regarding liver cancer, Embade et al. [187], reported significantly higher HuR protein levels in the mouse liver progenitor 29 (MLP29) cell line and in the S-adenosylmethionine-deficient (SAMe-D) cell line, isolated from the *methionine adenosyltransferase (MAT)1A* knockout (*MAT1A*-KO) mouse model of HCC, compared with primary mouse hepatocytes. Accordingly, higher HuR expression levels were found in human hepatoma cells (e.g., HepG2, Hep3B, SNU398, SNU449, SNU182, and SNU475), than in normal CRL4020 cells [188]. Moreover, in a human liver cancer tissue microarray (TMA) of 59 liver tissue cores from 44 patients, also by Zhu et al. [188], HCC tumor tissues showed significantly higher overall and cytoplasmic HuR staining, compared to normal liver tissues, and this high HuR staining score correlated with worse survival of patients with early-stage HCC. Furthermore, immunofluorescence analyses of normal versus malignant liver tissue revealed that HuR protein is down-regulated in normal human liver samples and up-regulated in HCC samples of different aetiologies (cirrhotic patients with HCV, alcoholic steatohepatitis, and non-alcoholic steatohepatitis (NASH)), where HuR concentration increased, proportionately, to their transformation status [189]. Finally, the expression of *ELAVL1* gene is highly induced in the tumor tissue of a cohort of CCA patients, according to The Cancer Genome Atlas (TCGA) mRNA expression repository [190]. Likewise, high cytoplasmic HuR levels are associated with poor survival in patients with surgically resected CCA, treated with adjuvant gemcitabine-based chemotherapy [191].

In sum, HuR overexpression, along with its cytoplasmic localization, are hallmarks of both HCC and CCA, correlating with disease progression and overall survival. Even though the role of HuR in HCC has been widely investigated, with some studies revealing HuR targets and regulators under these conditions, the function of HuR in CCA remains rather unexplored to date.

### 2.3. Signaling Pathways Implicated in HCC Involving HuR

**Figure 2:**
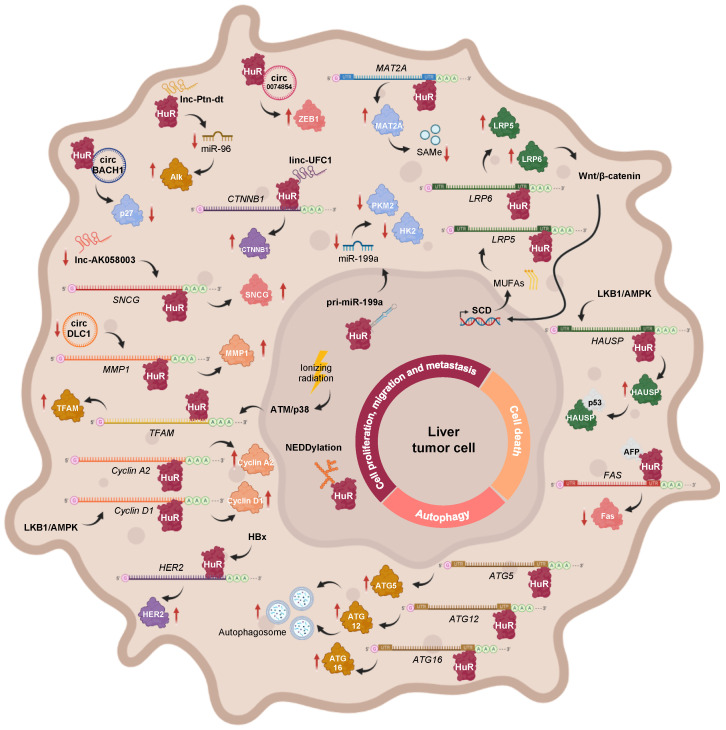
Main regulatory axes involving HuR, which are associated with cell proliferation, invasion, metastasis, apoptosis, and autophagy during HCC. These include the transcriptional, post-transcriptional, and post-translational modulators of HuR function, in addition to HuR target transcripts.

The oncogenic-gene-expression programs that allow cancer cells to develop, survive, proliferate, and colonize other tissues are strongly dependent on post-transcriptional mechanisms [28]. As has been previously described, HuR is highly involved in many types of cancer, including HCC and CCA, and so are the numerous HuR-regulated RNAs, which are known to contribute to the main cancer hallmark functions (i.e., enhanced cell proliferation and survival, elevated local angiogenesis, evasion of immune recognition, facilitated cancer cell invasion, and metastasis). Furthermore, a series of transcriptional, post-transcriptional, and post-translational regulators of HuR function have been found to be altered during HCC. Herein, we aimed to analyze the main regulatory axes involving HuR that are associated with hepatobiliary tumors, which can be classified into those related with cell proliferation, invasion, and metastasis; apoptosis; and autophagy (Figure 2). In the process, some of the previously reported HuR RNA targets and regulators of HuR function have been confirmed in the specific context of HCC, while others have been newly identified in this type of tumor.

#### 2.3.1. The Role of HuR in Cell Proliferation, Migration, and Metastasis during HCC

##### Pathways Involving Coding Transcripts

The PI3K/AKT signaling route is known to be upregulated in HCC [192], and, therefore, it could be responsible for the increased expression of the long HuR mRNA isoform, through NF-kB, as it occurs in gastric tumors [119]. However, it is likely that expression of the short HuR transcript is not observed during HCC, as reduced levels of BMP-7 and p-Smad1/5/8 were detected in patient samples [193].

During HBV-associated HCC, the HBV-encoded X (HBx) protein upregulates HuR expression, which enhances *HER2* mRNA stabilization and translation, thus contributing to the migration of HCC cells [194]. In the *MAT1A* knockout (*MAT1A*-KO) mouse model, which shows a chronic deficiency in SAMe levels and, spontaneously, develops NASH and HCC, hepatic levels of LKB1 and AMPK are activated, incrementing the cytoplasmic localization of HuR, which leads to the stabilization and expression of *cyclin A2* and *D1* mRNAs, and subsequent cell cycle progression [195]. Moreover, an abnormally low ratio between methylated and unmethylated HuR was revealed in HCC samples. The two HuR isoforms can associate with the 3′-UTR of *MAT2A* mRNA, whose activity is linked to liver cell proliferation. However, while unmethylated HuR was shown to stabilize the *MAT2A* transcript, an increase in its methylation status correlated with lower *MAT2A* mRNA levels. Hence, the loss of HuR methylation may explain the increased *MAT2A* mRNA and protein expression, and the subsequent loss of SAMe homeostasis that occurs during hepatocyte dedifferentiation, proliferation, and carcinogenesis [189]. Moreover, related with PTMs, NEDDylation of HuR was firstly reported in the context of liver cancer. Specifically, the E3 ligase Mdm2 catalyzes the conjugation of NEDD8 to HuR at Lys283, Lys313, and Lys326, a process that has been linked to the nuclear localization and reduced proteasomal degradation of the RBP [187]. Interestingly, the Mdm2 transcript being a described target of HuR [43,44], and considering the significantly positive correlation between Mdm2 and HuR expression in clinical HCC and human hepatoma cell lines [187], it would be highly expected that the mRNA levels of this E3 ligase were stabilized by HuR during HCC too, despite not having been verified to date.

The Wnt/β-catenin pathway has been shown to induce stearoyl-CoA desaturase (SCD) expression in liver-tumor-initiating and HCC cells, further increasing the synthesis of mono unsaturated fatty acids (MUFAs). MUFAs can block the nuclear import of HuR, thereby increasing its protein levels in the cytoplasm, where it binds to the 3′UTR of *Lrp5* and *Lrp6* mRNAs as well as stabilizes and stimulates their translation, further providing a positive feedback loop, by amplifying Wnt/β-catenin signaling and contributing to liver carcinogenesis [196]. Moreover, Wilms tumor 1-associated protein (WTAP) drives N6-methyladenosine (m6A) RNA methylation and epigenetic silencing of ETS1, by interfering with HuR-mediated stabilization of *ETS1* mRNA, further alleviating the expression of p21 and p27 G2/M checkpoint proteins, which are known downstream effectors of ETS1, and facilitating HCC progression [197]. Moreover, it has been reported that ionizing radiation activates the DNA damage response (DDR) via ATM/p38, which causes HuR shuttling to the cytoplasm in order to stabilize *mitochondrial transcription factor A (TFAM)* mRNA and induce its expression in HepG2 hepatoma cells. These results suggest a new pathway, which could be targeted to increase the sensitivity of liver cancer cells to radiotherapy [198].

##### Pathways Involving Non-Coding Transcripts

In addition to mRNAs, a few examples, whereby HuR interacts with non-coding transcripts during hepatobiliary tumors, have been reported. In human HCC cells subjected to hypoxic stress, HuR binds to the primary transcript of miR-199a (pri-miR-199a) blocking its processing into mature miR-199a. Interestingly, miR-199a is a negative regulator of *Hk2* and *Pkm2* mRNA expression. Therefore, HuR-mediated miR-199a maturation inhibition, during hypoxia, enables the metabolic reprogramming of HCC cells towards the Warburg effect, which confers favorable conditions for tumor growth, invasion, and metastasis [199]. The long intergenic noncoding RNA (lincRNA)-UFC1 plays an oncogenic role in liver cancer, by interacting with HuR, which stabilizes and induces the expression of the *CTNNB1* mRNA, leading to increased cell-cycle progression as well as proliferation and reduced apoptosis in HCC cells [200]. The oncofetal lncRNA Ptn-dt appeared to be highly expressed in HCC tissue and was found to interact with HuR protein, further compromising the stabilization and expression of miR-96. Therefore, the reduced function of miR-96 on the post-transcriptional inhibition of anaplastic lymphoma kinase (Alk) protein contributed to HCC cell proliferation [201]. Another study, describing the tumor suppressor role of lncRNA-AK058003 in HCC, revealed *SNCG* mRNA as a potential target of HuR. It was postulated that lncRNA-AK058003 is downregulated during HCC but, if overexpressed, it can interact with HuR to suppresses its expression, further affecting SNCG translation and stability, thus inhibiting γ-synuclein-mediated HCC cell proliferation and metastasis, both in vitro and in vivo [202].

In an attempt to elucidate the function of circRNAs, a few circRNA-RBP-mRNA axes involving HuR were revealed in HCC. For example, circBACH1 acts as an oncogene during hepatic tumorigenesis, upon association with HuR, to facilitate its translocation to the cytoplasm, where the RBP inhibits p27 protein expression and allows cell cycle progression, eventually favoring HCC cell proliferation [203]. KIAA1429, a key component of the m6A methyltransferase complex, negatively regulates circRNA-DLC1 in HCC tissues. A mechanistic study revealed that circDLC1 competitively binds with HuR, thereby impairing HuR-mediated *MMP1* mRNA stabilization and expression, ultimately resulting in decreased hepatoma cell proliferation and metastasis [204]. Moreover, hsa_circ_0074854 physically interacts and stabilizes HuR protein in the cytoplasm, which induces ZEB1 protein expression, thereby promoting the migration, invasion, and epithelial-mesenchymal transition (EMT) of HepG2 hepatoma cells [205].

#### 2.3.2. The Role of HuR in Cell Death during HCC

In SAMe-D cells derived from *MAT1A* KO mice, sustained LKB1 phosphorylation contributes to increased cytoplasmic HuR localization, where it, specifically, binds to the 3′-UTR of *herpesvirus-associated ubiquitin-specific protease (HAUSP)* mRNA, stabilizing it and increasing its transcription. The subsequent accumulation of HAUSP deubiquitinating enzyme in the cytoplasm allows its interaction with p53, which increases the stability of the tumor suppressor in the cytoplasm, thereby controlling the apoptotic response [206].

A recent study about a possible model, whereby alpha fetoprotein (AFP) regulates HCC progression and chemosensitivity, reported the reactivation of AFP during hepatocarcinogenesis and its interaction with HuR, resulting in the redistribution of the RBP to the cytoplasm. There, HuR would bind to the 3′-UTR of the Fas death receptor mRNA and repress its translation, without affecting its stability or splicing, which further suppresses the Fas/FADD-mediated extrinsic apoptotic program and bypasses immune surveillance in HCC-derived cell lines [188,207].

#### 2.3.3. The Role of HuR in Autophagy during HCC

Interestingly, HuR binds to the 3′-UTR of *ATG5*, *ATG12*, and *ATG16* mRNAs and enhances their translation. As a result, autophagosome formation is enhanced, dysregulating the autophagy activity in HCC cell lines, which might possibly act as a pro-survival response and promote hepatic tumor growth [208]. On the other hand, inhibition of autophagy by *BECN1* siRNA leads to HuR-enhanced ferroptosis in HCC. HuR and *BECN1* interaction induces autophagosome formation, increasing autophagic ferritin degradation and enhancing ferroptosis in hepatic stellate cells (HSCs) [209].

## 3. Therapeutic Approaches to Inhibit HuR in Hepatobiliary Tumors

Given its essential role as a regulation hub of cell-fate decisions, suppression of HuR activity has become a key objective to control tumor progression and therapy resistance. Even though RBPs were, initially, considered unsuitable for drug screening, developments during the last decade have enabled the generation of distinct approaches to target RBPs, including HuR, in cancer and other human pathologies [210].

The downregulation of HuR function may be achieved through different strategies, with small molecules being the most popular class of inhibitors for this RBP. Small molecules, directly, inhibit HuR interaction with target RNAs, oligomerization, nucleocytoplasmic shuttling, or post-translational modification, further compromising its function. Dehydromutactin, MS-444, and okicenone are three low-molecular-weight molecules, found to inhibit both HuR dimerization and RNA binding, during a screening of compounds derived from *Actinomyces* cultures [172]. Among them, MS-444 showed the highest affinity and has proven to affect the viability of different cell lines [173,211,212,213]. Dihydrotanshinone-I (DHTS) [214], b40, and quercetin [215], as well as the coumarin derivative CMLD-2 [216,217,218], were, also, identified as chemical disruptors of the interaction between HuR and its target mRNAs. Other small-molecule inhibitors preventing HuR interaction with its target mRNAs include KH-3, which has been found to be promising for the treatment of breast cancer [219]. A new inhibitor is the muscone derivative ZM-32, a synthetic compound that shows antiangiogenic effects in breast cancer tumor cells, by blocking HuR association with *VEGF* and *MMP9* mRNAs [220].

Except for ZM-32, which was tested in the human hepatoma HepG2 cell line without obtaining very successful outcomes [220], surprisingly, none of the abovementioned molecules have been tested in hepatobiliary tumors. Currently, studies reporting the use of small-molecule HuR inhibitors to tackle HCC are limited (Table 1). For instance, resveratrol (RSV) increased HuR mRNA and protein-expression levels in human liver cancer cell lines, which helped to raise *MAT2B* and *SIRT1* expression, by stabilizing their mRNAs at the 3′-UTR. Importantly, the induced HuR, SIRT1, and MATβ proteins interact and stabilize each other, while compromising the binding of MATβ to MATα2. Eventually, this leads to an increase in SAMe levels, which might favor cell growth suppression and apoptosis during liver cancer, upon treatment with RSV [221]. In another study, *N*-Benzylcantharidinamide impaired HuR translocation to the cytosol and decreased the stability of *MMP-9* mRNA and its expression, further inhibiting the invasive potential in metastatic Hep3B cells [222]. Additionally, since the nucleocytoplasmic transport of HuR depends on the interaction of this RBP with cytoskeletal proteins, the naturally occurring cytoskeletal inhibitors latrunculin A and blebbistatin have shown to exert antitumorigenic properties in human hepatoma cells, by interfering with the intracellular trafficking of HuR and its mRNA cargo [223]. Finally, the NEDDylation inhibitor Pevonedistat was shown to exert antitumoral effects in vitro and in vivo in liver cancer, partially through HuR destabilization. Importantly, overexpression of HuR in hepatoma cells offered resistance to pharmacological NEDDylation inhibition, while low levels of HuR sensitized cells to the treatment, suggesting that HuR levels determine the druggability of the NEDDylation pathway in HCC [224].

Notably, antisense oligonucleotides (ASOs) [225] and small interfering RNAs (siRNAs) exist for HuR. The latter have been widely used in the numerous studies mentioned throughout this review, in connection with HCC [187,188,189,194,197,199,204,206,208]. Related with siRNA technology as well, targeted delivery is, often, highly desirable and can be achieved thanks to the use of nanotechnology, for example, by means of the folate-receptor-targeted nanoparticle delivery of HuR siRNA, to reach lung cancer cells [226].

## 4. Future Perspectives

Herein, we have reviewed the current knowledge on the role of HuR in hepatobiliary tumors, including HCC and CCA. Considering increased HuR protein expression, cytoplasmic localization, and its relationship with patient outcome, HuR is regarded as a valuable diagnostic as well as a prognostic marker in HCC. Even though we recognize the growing attention of liquid biopsy in HCC, only invasive approaches have been carried out, to address the diagnosis and prognosis of HuR in these types of tumors, to date. With regard to CCA, high cytoplasmic expression and elevated tumor-gene levels of HuR have been described. However, special care should be taken when using cytological and pathological approaches, due to the highly desmoplastic nature of these types of tumors. In fact, in biliary tract carcinomas, biopsy samples are often inadequate for molecular profiling. Additionally, tissue sampling has reported high specificity but low sensitivity in diagnosis. Thereby, to further validate the relevance of HuR as a diagnostic and prognostic marker in CCA, further in vitro studies and in vivo pre-clinical mouse models are required.

Even though the role of HuR in CCA remains, largely, unexplored, novel mechanisms underlying HuR regulation have been addressed in HCC, with special focus on the importance of the PTM NEDDylation in the stabilization of HuR, preventing it from proteasome-mediated degradation [187]. Consequently, the relevance of HuR in pathological conditions, including cancer, has gained interest, with the advent of many naturally occurring and synthetic inhibitors of HuR. Nevertheless, given the lack of studies, using the currently available HuR inhibitors for the treatment of hepatobiliary tumors, and considering the heterogenous nature of HuR as a target affecting multiple pathways in many types of human cancers, it is, somehow, expected that the inhibition of HuR may be associated with undesired side-effects. Indeed, it has been described that HuR whole-body knock-out leads to embryonic lethality in mice, suggesting that HuR is, most probably, involved in regulating the fate of mRNAs encoding proteins implicated in key processes, such as organ development and tissue homeostasis [227].

Therefore, to take advantage of HuR as a therapeutic target, we propose that it would be critical to, specifically, inhibit the diverse interactions of HuR with its transcripts, which are uniquely relevant in the context of hepatobiliary cancers. For this purpose, the first step would be to interrogate the HuR RNA-binding signature in hepatobiliary tumors, which could be achieved, for example, by using RNA sequencing (RNA-Seq) approaches after the immunoprecipitation (IP) of the RNAs interacting with HuR, in the tumor and adjacent tissues, of large cohorts of HCC and CCA patients. Additionally, considering the relevance of HuR PTMs in liver disease, the screening of HuR PTM signature, including the residues susceptible of modification in liver cancer can unravel new unexpected and druggable regulatory mechanisms of HuR function. The screening of novel HuR RNA targets and PTMs in liver cancer as well as their clustering, according to the degree of liver disease, may, eventually, reveal subtypes of patients where certain HuR-related therapeutic approaches may be more suitable, paving the way for a more-personalized medicine. In other words, from this population-based study, we could, potentially, treat liver cancer by inhibiting the interactions of HuR either with (1) specific target RNAs or (2) peptides responsible for its PTM, which are ascribed to liver cancer. Indeed, short RNAs that compete with HuR for the binding to its target mRNAs, without affecting other HuR-mRNA binding interactions, had been previously suggested as a novel strategy for the management of HCC [228]. In this context, aptamers have, recently, emerged as small single-stranded DNA or RNA molecules that fold in a specific way, which is optimal to interact with a given target [229]. Thus, after elucidating HuR RNA targets and its PTMs in the context of liver cancer, we believe it would be possible to obtain aptamers blocking HuR interaction with both RNA and the peptides associated with PTMs, in a highly specific manner, minimizing potential adverse effects. This might be achieved through the screening of aptamer libraries, using the Systematic Evolution of Ligands by Exponential Enrichment (SELEX) selection method. For this purpose, the understanding of HuR protein structure is of great benefit to predict whether the conformation of the aptamer would be suitable to inhibit the binding of HuR to its target.

## 5. Concluding Remarks

In conclusion, even though, in the past few years, great efforts have been carried out, in order to better understand the function of HuR and its regulation in hepatobiliary tumors, further studies are necessary to address the therapeutic potential of HuR inhibitors or, alternatively, a more-personalized treatment based on the selected inhibition of HuR and its RNA or the protein targets in HCC and CCA.

## Figures and Tables

**Table 1:** Small-molecule inhibitors controlling HuR function, tested in hepatobiliary tumors.

Molecule	Type	Effect	Ref.
ZM-32	Synthetic muscone-derivative	Inhibition of HuR binding to target mRNAs	[220]
Resveratrol (RSV)	Naturally occurring	Increase in HuR mRNA and protein expression	[221]
*N*-Benzylcantharidinamide	Synthetic analogue of the naturally occurring cantharidine drug	Inhibition of HuR translocation to the cytosol	[222]
Latrunculin A	Naturally occurring	Inhibition of HuR translocation to the cytosol	[223]
Blebbistatin	Naturally occurring	Inhibition of HuR translocation to the cytosol	[223]
Pevonedistat	Synthetic NEDDylation inhibitor	HuR destabilization	[224]
